# Loxapine and Cyproheptadine Combined Limit Clozapine Rebound Psychosis and May Also Predict Clozapine Response

**DOI:** 10.1155/2016/6123913

**Published:** 2016-06-28

**Authors:** Lila Aboueid, Richard H. McCarthy

**Affiliations:** ^1^SUNY Downstate Medical Center, Department of Psychiatry, 450 Clarkson Avenue, Brooklyn, NY 11203, USA; ^2^Kingsboro Psychiatric Center, 681 Clarkson Avenue, Brooklyn, NY 11203, USA

## Abstract

Clozapine has been consistently shown to be superior to other antipsychotics in the treatment of psychosis. However, clozapine usage has been limited due to required routine blood monitoring and the potential for life threatening side effects. We report a case of a 66-year-old female patient, who developed clozapine-induced agranulocytosis after 10 weeks of clozapine treatment and was subsequently successfully treated with a combination of loxapine and cyproheptadine. The combination is thought to mimic the pharmacological profile of clozapine, rendering it as a possible alternative to traditional clozapine treatment.

## 1. Introduction

Clozapine, the only FDA approved antipsychotic used in treatment refractory schizophrenia and suicidality, has consistently been shown to be more effective than any other antipsychotic medication. However, clozapine-induced agranulocytosis limits clozapine's use, and when it occurs, clozapine must be stopped and cannot be restarted. This not only results in the loss of incremental benefits that clozapine afforded, but also may lead to particularly severe, difficult to treat rebound psychosis. Cyproheptadine has been shown to limit this rebound. Cyproheptadine, an atypical serotonin 2 antagonist, mimics some of clozapine's actions [1]. In addition to softening clozapine discontinuation symptoms, cyproheptadine, like clozapine, decreases negative symptoms [2], increases appetite and weight gain [3], and improves extrapyramidal symptoms (EPS) and motor symptoms [4].

Additionally, loxapine, a dibenzoxazepine tricyclic antipsychotic, with a chemical structure very similar to clozapine, is proposed to be mediated through high-affinity antagonism of postsynaptic dopamine D2 receptors and serotonin 5-HT2A receptors. Loxapine has not only been shown to be effective in the treatment of schizophrenia, but has recently been approved for the treatment of mild-moderate agitation in adults with schizophrenia or bipolar disorder available for inhalational administration [5].

There has been considerable speculation about clozapine's exact mechanism of action, but it remains unknown. Using PET data Kapur and Zipursky reasoned that a loxapine and cyproheptadine combination could have a 5HT-2A/D2 ratio, D4, antihistamine, and antimuscarinic receptor blockade profile similar to clozapine [1]. Receptor blockade studies by others also support this [6]. The combination of cyproheptadine and loxapine may mimic clozapine's mechanism of action and, thus, is postulated as an alternative to traditional clozapine usage.

## 2. Case Presentation

This is the case of a 66-year-old Caucasian female, with a long history of treatment of refractory schizophrenia. In spite of multiple antipsychotic medication trials and current medication regimen (lithium 450 mg daily, quetiapine 300 mg daily, and gabapentin 200 mg daily), the patient's paranoid delusions had not abated. Her daily life was increasingly impaired by her delusions and she was referred for a clozapine trial (started 5/12/14). Clozapine was titrated to a maximum daily dose of 300 mg daily, lithium was continued at 450 mg daily, and quetiapine and gabapentin were titrated off and discontinued (see Table 1). The patient had a rapid and significant decrease in her delusions, documented early in the clozapine trial, which continued to abate with subsequent dose increases.

**Figure 1:**
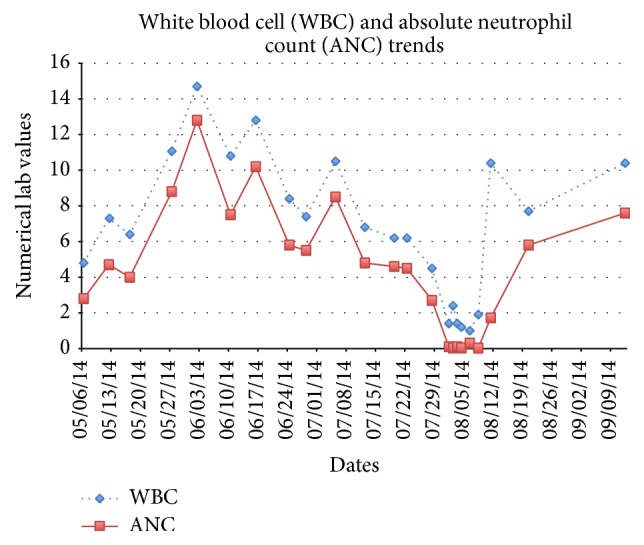


Initially, hypotension limited dose increases, but at ten weeks she had a precipitous drop in her white blood cells (WBC) and absolute neutrophil count (ANC) (Figure 1) that proceeded to full blown agranulocytosis over a three-day period. Filgrastim treatment was begun; clonazepam was used to contain anxiety and the patient was started on cyproheptadine 4 mg TID to prevent clozapine discontinuation rebound psychosis. When the patient's hematological indices returned to normal, the patient was begun on loxapine 10 mg daily to address her newly returned paranoid delusions. Over the next three weeks, the patient's delusions continued to decrease to levels lower than they had been on clozapine. At this time, about two years after clozapine discontinuation, the delusions are only minimally present and do not result in any interference in the patient's daily life.

## 3. Discussion

Clozapine is well known for its efficacy in patients with treatment of resistant schizophrenia. Although clozapine may be clinically effective, the treatment may be stopped abruptly due to patient nonadherence, side effects (i.e., neutropenia/agranulocytosis), and/or medical complications (i.e., stroke, seizure, pulmonary embolism, etc.). Possible consequences of abrupt clozapine discontinuation include the rapid onset of an unusually severe psychotic relapse that is largely unresponsive to other treatments [7], the psychotic relapse following clozapine's rapid discontinuation can be prolonged [8], and/or the patient may not respond to clozapine when it is reintroduced in the future [9]. In some cases, clozapine discontinuation may result in delirium [10], severe anxiety, altered levels of consciousness, mental status changes, insomnia [11], somatic complaints including nausea, vomiting, diaphoresis, akathisia, motor restlessness, and dyskinetic movements, and excited catatonia and/or neuroleptic malignant syndrome (NMS) [12] that may cloud the clinical picture.

Clozapine, along with other atypical psychotics, fundamentally differs from typical antipsychotics in that they block a high number of serotonin receptors and lower number of D2 receptors. Given this pharmacological profile, it is reasonable to assume other agents with similar properties may be combined in effort to treat psychosis. However, the dose of the antipsychotic should be low enough to provide a low level of D2 blockage (less than 80% occupancy) to retain the dopamine and serotonin blockage. Based on previous studies, 15 mg/day of loxapine would result in lower than 80% receptor occupancy (typical dosage used is 25–100 mg/day, where D2 occupancy is greater than 80%). Cyproheptadine has been known to block 85–95% of serotonin 2 receptors in humans with doses of 12–18 mg/day. Thus, the combination of the two medications has been formulated to yield a similar pharmacological profile seen with clozapine, as well as other atypical antipsychotics [1].

The combination of loxapine and cyproheptadine mimics some of clozapine's action. In cases where a clozapine responder must discontinue the medication, there may be an alternative to clozapine's use. In addition, patients reluctant to take clozapine may be offered this combination to determine if clozapine or this combination may benefit them. Further assessment for the use of loxapine and cyproheptadine is indicated.

## Figures and Tables

**Table 1:** 

Date	WBC	ANC	Clozapine (mg/day)	Lithium (mg/day)	Quetiapine (mg/day)	Gabapentin (mg/day)	Clonazepam (mg/day)	Cyproheptadine (mg/day)	Loxapine (mg/day)
05/06/14	4.8	2.8		**450**	300	200	0.5 qid prn		
05/12/14	7.3	4.7	12.5	450	300	200	0.5 qid prn		
05/12/14	7.3	4.7	37.5	450	300	200	0.5 qid prn		
05/17/14	6.4	4	100	450	300	200	0.5 bid prn		
05/27/14	11.1	8.8	50	450	300	200	0.5 bid prn		
06/02/14	14.7	12.8	50	450	300	200	0.5 daily prn		
06/10/14	10.8	7.5	100	450	300	200	0.5 daily prn		
06/16/14	12.8	10.2	150	450	300	100	0.5 daily prn		
06/24/14	8.4	5.8	150	450	250	0	0		
06/28/14	7.4	5.5	150	450	200				
07/05/14	10.5	8.5	150	450	50				
07/12/14	6.8	4.8	125	450	50				
07/19/14	6.2	4.6	150	450	0				
07/22/14	6.2	4.5	200	450					
07/28/14	4.5	2.7	300	450					
08/01/14	1.4	0.1		450				16	
08/02/14	2.4	0.029		450			0.5 bid	16	
08/03/14	1.4	0.1		450			0.5 bid	16	
08/04/14	1.2	0.03		450			0.5 bid	16	
08/04/14	1.2	0.03		450			0.5 bid	16	
08/06/14	1	0.3		0			0.5 bid	16	
08/08/14	1.9	0.021					0.5 bid	16	
08/11/14	10.4	1.72					0.5 bid	16	10
08/20/14	7.7	5.8					0.5 bid	16	10
09/12/14	10.4	7.6					0.5 bid	16	10
